# Growth differentiation factor 5 in cartilage and osteoarthritis: A possible therapeutic candidate

**DOI:** 10.1111/cpr.12998

**Published:** 2021-02-01

**Authors:** Kai Sun, Jiachao Guo, Xudong Yao, Zhou Guo, Fengjing Guo

**Affiliations:** ^1^ Department of Orthopedics Tongji Medical College Tongji Hospital Huazhong University of Science and Technology Wuhan China

**Keywords:** cartilage development, cartilage homeostasis, chondrocyte, GDF‐5, osteoarthritis

## Abstract

Growth differentiation factor 5 (GDF‐5) is essential for cartilage development and homeostasis. The expression and function of GDF‐5 are highly associated with the pathogenesis of osteoarthritis (OA). OA, characterized by progressive degeneration of joint, particularly in cartilage, causes severe social burden. However, there is no effective approach to reverse the progression of this disease. Over the past decades, extensive studies have demonstrated the protective effects of GDF‐5 against cartilage degeneration and defects. Here, we summarize the current literature describing the role of GDF‐5 in development of cartilage and joints, and the association between the *GDF‐5* gene polymorphisms and OA susceptibility. We also shed light on the protective effects of GDF‐5 against OA in terms of direct GDF‐5 supplementation and modulation of the GDF‐5‐related signalling. Finally, we discuss the current limitations in the application of GDF‐5 for the clinical treatment of OA. This review provides a comprehensive insight into the role of GDF‐5 in cartilage and emphasizes GDF‐5 as a potential therapeutic candidate in OA.

## Graphical abstract

GDF‐5 is essential for cartilage development and homeostasis. *GDF‐5* gene polymorphisms are significantly associated with OA susceptibility. Supplementation of GDF‐5 and regulation of its expression exhibit prominent curative effects in experimental OA and cartilage defects. GDF‐5 may be a novel potential therapeutic agent for OA.

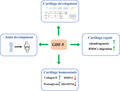

## 1 INTRODUCTION

Cartilage is an integral part of the skeletal system that is composed of specialized cells, primarily chondrocytes, which have critical functions in skeletal development, tissue patterning, secreting the cartilage matrix and maintaining the normal activity of joints.^1^ Cartilage development involves chondrogenesis, which starts with mesenchymal stem cell (MSC) condensation. Following this, chondrocytes are derived from the chondrogenic differentiation of MSCs. The resting chondrocytes secrete an abundant extracellular matrix (ECM) mixture of aggrecan (ACAN) and collagen type II to form a cartilage template (anlagen).^1^, ^2^ The differentiated cells undergo the process of endochondral ossification through the steps of chondrocyte proliferation and maturation into hypertrophic chondrocytes. Ultimately, these terminally differentiated chondrocytes undergo apoptosis and are replaced by the bone at the primary ossification center.^3^ Subsequently, the secondary ossification centre newly develops at the epiphysis and radially spreads within it. The part between the secondary ossification centre and the joint cavity is permanently retained as articular cartilage.^4^ Articular cartilage is a highly specialized tissue that covers and protects the ends of long bones. It consists of collagen type II, hyaluronic acid, and a rich proteoglycan matrix secreted by chondrocytes.^5^ These cells regulate the homeostasis of articular cartilage through balancing their own anabolism and catabolism.^6^ Because of the unique function of cartilage, the interference during chondrogenesis and the loss of cartilage tend to cause joint disorder.

Osteoarthritis (OA) is one of the most common joint diseases, primarily owing to the progressive destruction of cartilage.^7^ In addition, features of OA include synovial inflammation and subchondral bone remodelling.^7^ The disease severely influences people's quality of life worldwide, and the incidence of OA is increasing because of the ageing population throughout the world.^7^ Nevertheless, current treatment options for OA are limited to relieving pain and promoting functional improvement in patients rather than inhibiting OA progression. Therefore, there is an urgent need to develop more effective pharmacologic treatments for OA. Growth factors are a group of bioactive endogenous polypeptides that can stimulate cellular growth, proliferation and differentiation.^8^ In articular cartilage, numerous growth factors, such as transforming growth factor‐β1 (TGF‐β1), insulin growth factor I, bone morphogenetic protein (BMP) and fibroblast growth factors, have been widely investigated regarding the regulation of development and homeostasis of articular cartilage.^9^ In addition, growth factors are readily available and recombined with fewer side‐effects. Therefore, growth factors offer promising therapeutic approaches for OA. Among these growth factors, growth differentiation factor 5 (GDF‐5), a member of the TGF‐β family, has attracted wide attention in the field of OA. GDF‐5 is one of the earliest markers of joint development.^10^ The steps underlying joint development, growth, remodelling and homeostasis require regulation of GDF‐5 and relative signalling pathways.^11^ Therefore, this growth factor is critical to joint health. Accumulated evidence has revealed a robust and highly reproducible correlation between *GDF5* and knee OA susceptibility.^12^ *GDF‐5* deficiency contributes to the pathogenesis of OA,^13^ whereas GDF‐5 supplementation has a beneficial effect on the experimental model of OA.^14^ These studies raise the possibility of manipulating GDF‐5 levels for the treatment of OA. In this review, we will provide an overview of GDF‐5 and its roles in cartilage development and OA. Importantly, we highlight and discuss the potential applications of GDF‐5 for OA treatment.

## 2 OVERVIEW OF GDF‐5

GDF‐5, also known as cartilage‐derived morphogenetic protein‐1 (CDMP‐1) or bone morphogenetic protein‐14, is a member of the TGF‐β/BMP superfamily.^15^ It was first identified in 1994,^16^ and subsequently, human GDF‐5 was cloned during the same year.^17^, ^18^ The structure of GDF‐5 is closely related to the BMPs.^19^ GDF‐5 is synthesized as a large precursor protein, which comprises two major domains: the N‐terminal prodomain with a cleavage site and signal sequence and the active C‐terminal domain. The precursor protein then is cleaved at a characteristic RXXR (Arg, X, X, Arg) site to release the active peptide. The active peptide is highly conserved with seven cysteine residues^19^, ^20^ and contains two regions: the N‐terminal region, which is involved in forming a tail‐like structure within GDF‐5 dimers, and the C‐terminal region, which is responsible for forming of homodimers and heterodimers.^21^

GDF‐5 transduces signals by binding to two types of the transmembrane serine/threonine kinase receptors, types I and II.^22^, ^23^ Among the seven known type I receptors, BMP receptor (BMPR)‐IA and BMPR‐IB have been demonstrated to be associated with skeletal patterning.^24^, ^25^, ^26^ GDF‐5 has high binding affinity to BMPR‐IB, BMPR‐II and Activin type II receptors.^26^, ^27^ Upon binding to type I and type II receptors, the signalling cascade of GDF‐5 activates the downstream Smad pathway. The phosphorylated Smad 1/5/8 then forms the complex with Smad 4, a common Smad that translocates into the nucleus to regulate the transcription of multiple genes including *COL2A1* and *ACAN,* and cellular processes, such as proliferation, differentiation and synthesis of the ECM.^28^, ^29^

## 3 GDF‐5 IN CARTILAGE DEVELOPMENT

Chondrogenesis is the differentiation process that leads to the formation of cartilage and bone.^30^ This process involves the recruitment, migration and condensation of mesenchymal cells as well as differentiation and maturation of chondrocytes.^31^ The *GDF‐5* expression profile indicates its importance in chondrogenesis, especially in prechondrogenic condensation.^32^

In vivo studies have shown that mutations in *Gdf‐5* may cause the autosomal recessive syndromes such as brachypodism (bp) in mice and Grebe‐type and Hunter‐Thompson chondrodysplasia in humans, characterized by the shortening of the skeletal elements and abnormal development of some joints.^24^, ^33^, ^34^ To investigate how GDF‐5 controls skeletogenesis, some in vivo molecular function studies have been conducted, and the results showed that overexpressed *Gdf‐5* in the embryos and developing chick limb increased the size of the skeletal elements during the condensation stage or later when the skeletal elements have been formed respectively.^35^ *Gdf‐5* overexpression can initiate chondrogenesis, which enhances cell adhesiveness of mesenchymal cells and proliferation of chondrocytes.^35^, ^36^

During mesenchymal condensation, the presence of GDF‐5 stimulated the activities of N‐cadherin and further improved cell‐cell adherence, thereby promoting condensation.^37^ In in vitro chondrogenic differentiation, MSCs are regularly investigated by using a pellet culture system in which MSCs are centralized to mimic mesenchymal condensation.^32^, ^38^ Supplementation of recombinant human GDF‐5 protein (rhGDF‐5) can significantly enhance bone marrow‐derived mesenchymal stromal cells (BMSCs) chondrogenic differentiation in chondrogenic MSC pellets, as evidenced by the increased incorporation of collagen type II and sulphated glycosaminoglycan (GAG) into the ECM. Furthermore, GDF‐5 promoted the transition of these cultures to hypertrophy and maturation.^39^, ^40^ In three‐dimensional aggregate culture, GDF‐5, TGF‐β1 and BMP‐2 supplementation each significantly upregulated chondrogenic gene expression in human MSCs. Intriguingly, the combination of GDF‐5, TGF‐β1 and BMP‐2 promoted the greatest upregulation of chondrogenic genes, including *SOX9*, *COL2A1* and *ACAN*, and synthesis of cartilage‐specific matrix, eventually yielding robust cartilage rich in GAGs and collagen type II after 4 weeks of maturation.^41^ In addition, recombinant GDF‐5 protein can also improve chondrogenic differentiation in other stem cells, such as foetal human MSCs,^42^ human embryonic stem cells,^43^ canine MSCs,^44^ embryonic chick mesenchymal cells^37^ and rabbit adipose‐derived stromal cells.^45^ Furthermore, adenovirus‐mediated overexpression of *Gdf‐5* exerted similar effects on chondrogenesis in adipose stem cells.^46^

The phosphorylation of Smad 1/5/8, the downstream signalling molecule of TGF signalling, is greatly involved in the terminal differentiation of chondrocytes.^47^ GDF‐5 treatment induced Smad 1/5/8 phosphorylation in chondrogenic MSC pellets,^39^ suggesting that these Smads contribute to the chondrogenic differentiation of these cultures and their hypertrophic differentiation.

In addition to Smads, p38 mitogen‐activated protein kinase (MAPK) and Trps1, a transcriptional regulator, also contribute to the effect of GDF‐5 on chondrocyte differentiation.^37^, ^48^ GDF‐5 enhanced nuclear translocation of Trps1 and phosphorylation of p38, and further increased *COL2A1* gene expression in the chondrogenic cell line ATDC5. These effects were blocked by SB203580, an inhibitor of the p38 signalling. Moreover, Trps1‐overexpressing ATDC5 cells were prone to differentiate into chondrocytes.^48^, ^49^ Taken together, Trps1 and p38 act as downstream of the GDF‐5 signalling pathway and promote the differentiation of chondrocyte.

**FIGURE 1:**
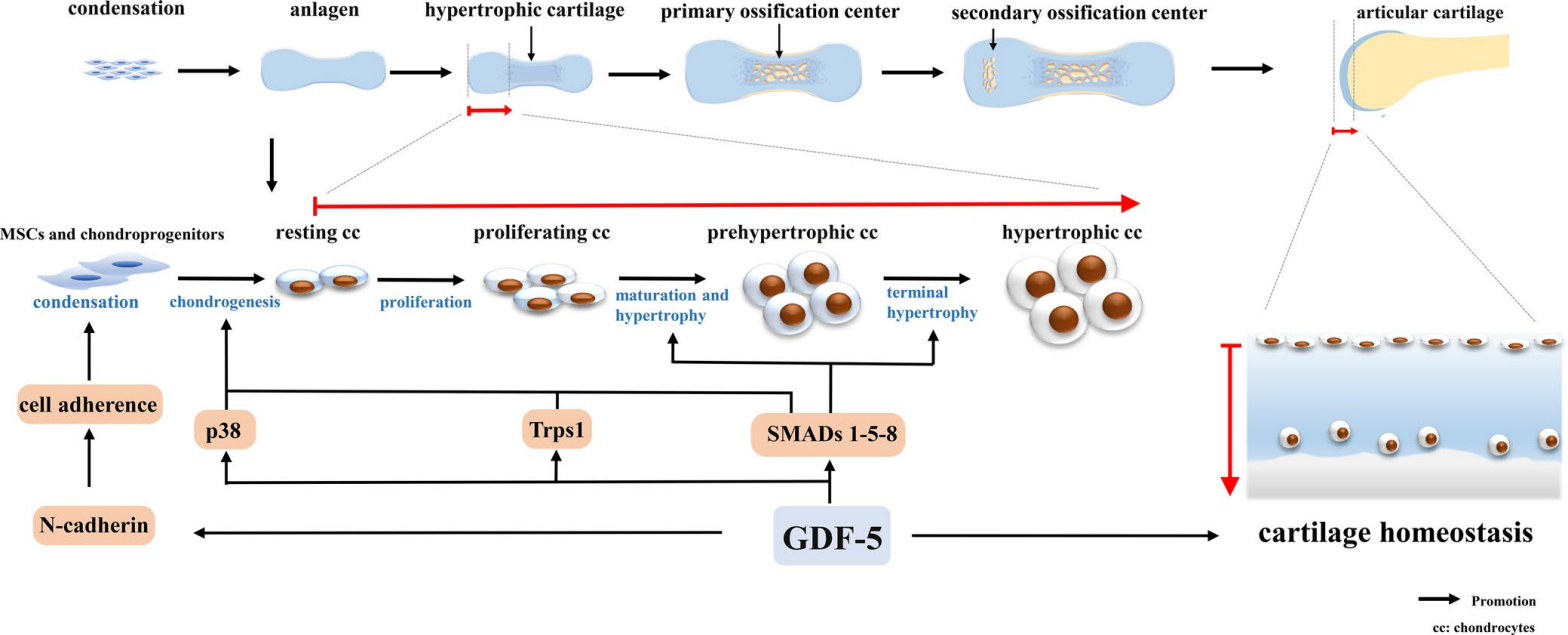
The role of GDF‐5 during cartilage development. The process starts with mesenchymal stem cell (MSCs) condensation. Condensed cells are differentiated into chondrocytes that construct a cartilage template (anlagen) by synthesizing ECM. Chondrocytes at the centre of condensation become hypertrophic and hypertrophic cartilage is calcified. These terminally differentiated chondrocytes undergo apoptosis and are replaced by the calcified bone in the primary ossification centre. Later, the secondary ossification centre is newly developed at the epiphysis and radially spreads within it. The part between the secondary ossification centre and the joint cavity is permanently retained as articular cartilage. During cartilage development, GDF‐5 promotes mesenchymal cell condensation and differentiation into chondrocytes and further stimulates chondrocytes differentiating into proliferative, prehypertrophic and hypertrophic cells. In articular cartilage, GDF‐5 functions to maintain cartilage homeostasis

All these findings demonstrate that GDF‐5 is essential for chondrogenesis, which governs the development of cartilage and bone (Figure 1).

## 4 GDF‐5 IN JOINT DEVELOPMENT

Over the past decades, the emergence of the interzone at the presumptive joint site has been considered as an initial indicator of joint development, and the interzone cells have been suggested to serve as joint progenitors.^50^ The interzone, derived from mesenchymal tissue, loses the specific gene expression of chondrocytes and, notably, expresses GDF‐5, which acts as an indicator of the interzone in the early development of joints.^51^, ^52^ It has been found that the gene mutation of the human *GDF‐5* and its receptor BMPR‐1B can lead to skeletal malformations, including brachydactyly and/or chondrodysplasia.^25^, ^33^, ^53^, ^54^ In addition, mice with brachypodism exhibit sufficient GDF‐5 expression occurring out of the interzone, giving rise to the fusion of digit joints.^55^ These findings confirm that GDF‐5 is critical for the development of joints.

In constitutively active *Gdf‐5*‐Cre mice, the broad labelling of R26R‐reporter cells was seen throughout multiple joint tissues.^56^ In another study, Decker et al^57^ mated *Gdf‐5*‐Cre and ROSA‐*LacZ*‐reporter mice to investigate the lineage of early GDF‐5‐expression and found that although *Gdf‐5* mRNA expression was highly diminished in joint tissues at birth, *Gdf‐5*‐Cre R26R *LacZ* (*Gdf‐5*‐Cre +) labelled cells were found in most mouse joint tissues, including the articular cartilage, meniscus, intra‐joint ligaments and synovial lining at maturity. This suggests that cells with a GDF‐5‐expressing lineage are not transient and actively participate in joint tissue formation.

**FIGURE 2:**
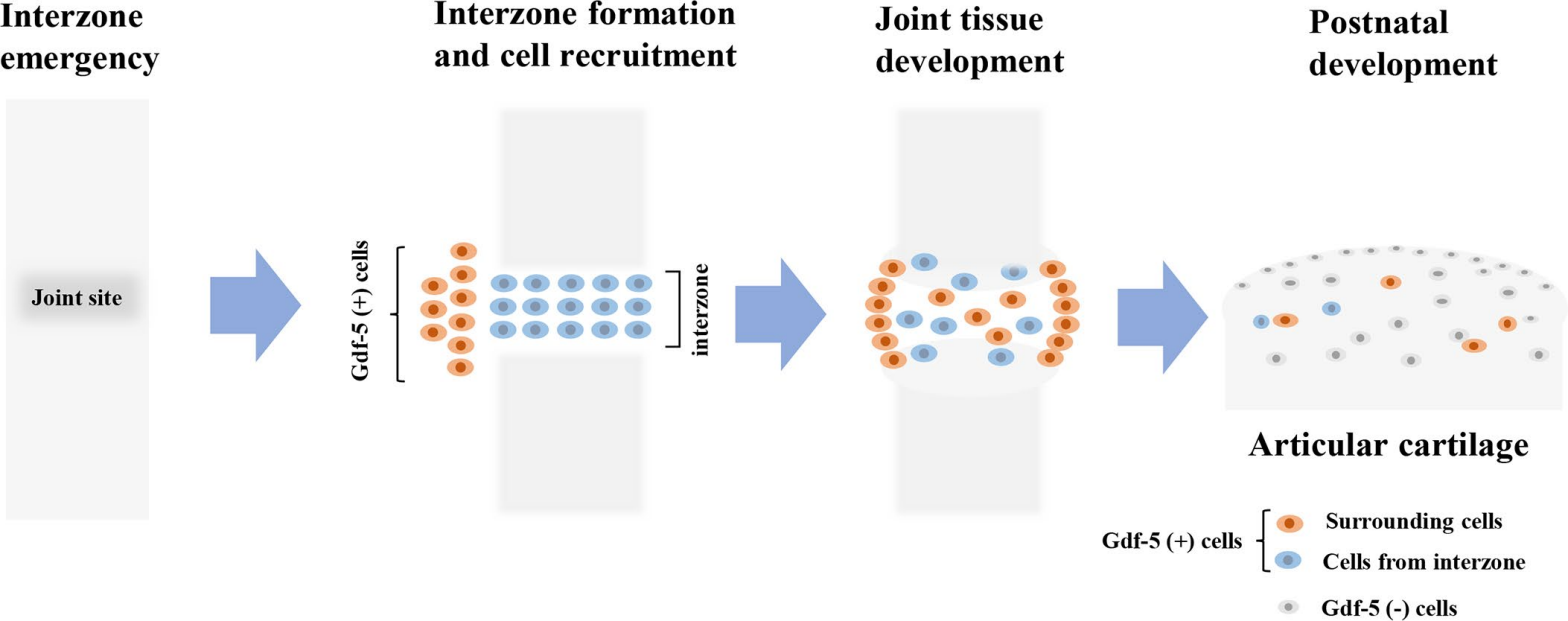
GDF‐5 (+) cells are involved in joint formation. The interzone emerges at the presumptive joint site within a pre‐cartilage tissue. Mesenchymal GDF‐5 (+) cells constitute the interzone area at incipient joint sites in early embryonic limbs, and flanking cells are recruited from surrounding tissues into the GDF‐5 lineage. Subsequently, joint cavitation occurs in the centre of the interzone, and the surrounding cells influx into the joint region. The GDF‐5 (+) cells in the interzone and its surroundings gradually form articular cartilage and synovial joints. In the late stage of joint development, GDF‐5 is decreased in the knee region and GDF‐5 (+) cells are restricted within the eventual articular cartilage

However, recent evidence has suggested that cells from the surrounding area are integrated to form joints during development.^56^, ^58^ In a study of Shwartz et al,^51^ GDF‐5‐positive cells were detected in the epiphyses, articular cartilage, meniscus and intra‐articular ligaments in the knee from E10.5 to E18.5. Furthermore, by using a knock‐in *Gdf‐5*‐CreERT2 and Cre‐dependent reporter mice, Shwartz et al^51^ found that GDF‐5 (+) cells from the surrounding tissues continuously flowed into the interzone, where they contributed to the formation of joint tissues. In contrast, the interzone/early‐specific cells lost GDF‐5 expression and migrated out to primarily form the epiphyseal cartilage. The similar migration was also observed by Decker et al.^59^ The major cells responsible for forming the interzone did not form articular cartilage but formed transient cartilage, meniscus and ligaments.^51^ On the other hand, the peripheral cells have been well‐recognized to prominently contribute to the formation of joint components.^4^ In the late stage of joint development, GDF‐5 expression was decreased in the knee region and appeared to be dispensable to joint development.^52^ Thus, the role of GDF‐5 in the joint/cartilage development may be determined by their spatiotemporal environment. In *Gdf5*‐CreERT2/R26‐zsGreen mice, the CreERT2‐positive cell labelling was more restricted to cells within the eventual articular cartilage after tamoxifen administration at late embryonic time points.^59^ These labelled cells and/or their progeny remained in the articular cartilage until at least 6 months of age^59^ (Figure 2). In adulthood, GDF‐5 might be related to homeostasis of the articular cartilage because genome‐wide association studies have revealed that *GDF‐5* is a susceptible gene for OA.^60^

## 5 GDF‐5 IN OA

### 5.1 *GDF‐5* gene polymorphism is associated with knee OA

Single‐nucleotide polymorphisms (SNPs) are common genomic DNA variations within a population.^61^ An SNP located within the coding sequence of a gene may give rise to an amino acid substitution and further alter protein function, which might be associated with susceptibility to human diseases.^61^ It is widely acknowledged that SNPs in some genes are related to the susceptibility to OA.^62^, ^63^ Such genes include those participating in signalling cascades involved in joint and bone biology as well as those involved in inflammatory pathways.^64^ SNPs in these genes are closely related to abnormal proliferation and differentiation of chondrocytes, leading to abnormal cartilage development and morphology.^65^ Over the past decades, numerous studies have demonstrated that polymorphisms in *GDF‐5* highly contribute to the pathogenesis of OA.^60^, ^66^, ^67^ In particular, the SNP rs143383, a T‐to‐C transition in the 5ʹ untranslated region (UTR) of the *GDF‐5* gene and the *GDF5* promoter, is a major susceptibility allele for OA in Asian and European populations.^60^, ^68^, ^69^ Expression of the OA‐associated T allele has been found to be significantly lower than that of the C allele in patients with OA.^68^, ^69^, ^70^ Also, a meta‐analysis based on 23 995 subjects has shown that the C allele of the GDF5 gene was protective for knee OA susceptibility across different populations.^71^ However, there are some differences in the association between genetic variants of *GDF‐5* and OA of the knee, hip and hand. Using a random effects model, a significant difference was identified between patients with knee OA and controls for the T allele of rs143383. For hand OA, a moderate association was observed for rs143383 in the combined population. However, non‐statistically significant summary odds ratio of hip OA was found in both combined studies and European studies.^72^ For age, significant differences between knee OA and the control group were found in individuals aged more than 60 years, and no significant difference was observed for those aged less than 60 years in Asian population.^73^

The imbalanced allelic expression could lead to reduced GDF‐5 expression in the whole joint, which probably increases individual susceptibility to OA.^74^ The trans‐acting factors Sp1, Sp3 and DEAF‐1 have been identified as transcriptional repressors of *GDF‐5* expression by modulating the allelic imbalance of rs143383.^74^ Furthermore, Reynard et al^55^ found that the modification of DNA methylation regulated the functional effect of the OA SNP (rs143383) and *GDF‐5* expression in cartilage. Specifically, the CpG sites formed by the C alleles of rs143383 were methylated in cell lines and joint tissues, and methylation of the proximal promoter and 5′ UTR resulted in decreased transcriptional activity of these regions. In contrast, demethylation of several CpG sites within the 5′‐UTR by treatment with 5‐Aza‐2′ deoxycytidine, a demethylating agent, was associated with increased expression of the C allele of rs143383 relative to the T allele and *GDF‐5* expression.^55^ Additionally, the altered binding of SP1 and SP3 repressor to rs143383 has been reported to mediate the regulatory role of CpG methylation in the allelic expression of *GDF‐5*.^75^ Hence, these findings suggest that DNA methylation accounted for the allelic imbalance of rs143383, as observed in OA joint tissues. Demethylating agents, such as decitabine, may have potential therapeutic effects on OA. Furthermore, Egli et al^70^ identified additional *GDF‐5* polymorphisms, rs143384 and 2250ct. The rs143384 in the 5′‐UTR region can affect the expression of *GDF‐5* rs143383. However, 2250ct, located in the 3′‐UTR, influenced the *GDF‐5* allelic expression independent of rs143383, although this effect was comparable to that observed for rs143383.

However, Shin et al^76^ reported that the *GDF‐5* SNP rs143383 was not associated with for primary knee OA in Korea. A similar outcome was reported by Tsuzou et al in another study performed with Greek Caucasians, and no significant differences in allelic and genotypic frequencies were found when the individuals were stratified by sex.^77^ The different results regarding rs143383 in different populations might be attributed to ethnic differences in *GDF‐5* methylation caused by environmental and genetic factors.^74^ The existence of regulatory polymorphisms highlights the complexity of the regulation of *GDF‐5* expression. Importantly, it also provides multiple sites to better understand the susceptibility to OA and develop possible preventive approaches.

### 5.2 The expression of GDF‐5 during OA

GDF‐5 is involved in the pathology of the whole joint organ, which could be reflected by altered expression of GDF‐5 at the different stages of OA. GDF‐5 expression has been found to be upregulated in the cartilage of patients with OA compared with that in control patients.^75^ Further, GDF‐5 expression was correlated with the expression of WNT9A and SOX11, which are known upstream regulators of GDF‐5 expression.^78^ In an experimental OA mice model that underwent surgical unilateral destabilization of the medial meniscus (DMM), GDF‐5 downstream regulatory elements were activated in articular chondrocytes at 2 weeks, particularly in areas of initial damage.^78^ Moreover, GDF‐5 was also highly expressed during cartilage repair in DMM mice and was switched on in injured synovium in the prospective areas of cartilage formation, which implies that GDF‐5 plays a role in cartilage repair.^78^ These outcomes suggest that GDF‐5 is upregulated and might play a positive role in the early phase of OA.

However, at 8 weeks after DMM, GDF‐5 expression was reduced in the areas of cartilage damage.^78^ Similarly, a study by Kan et al^79^ showed that SOX11 and GDF‐5 expression were decreased in degraded cartilage in mice that underwent transection of the medial collateral ligament and medial meniscus at 4 weeks. In addition, decreased GDF‐5 expression in cartilage in patients with OA was observed in studies by Bobinac D et al^80^ and Wu et al.^81^ Reduced GDF‐5 expression in OA cartilage might be attributed to the fact that most cartilage specimens were obtained from patients with late‐stage OA, who suffered from extensive damage. Moreover, GDF‐5 polymorphisms causing decreased expression contribute to susceptibility to OA.

Chronic low‐grade inflammation causes many pathologic changes in OA.^82^ This pathological condition is mediated primarily by inflammatory mediators, including tumour necrosis factor α (TNF) and interleukin 1β (IL‐1β).^82^ Bobacz K et al^83^ showed that GDF‐5 expression was also reduced in articular cartilage in TNF‐transgenic mice with chronic arthritis. Additionally, GDF‐5 was expressed in fibroblasts of adult synovial tissue and may counteract macrophage infiltration, which is considered an indicator of inflammatory infiltration. Its expression in the superficial lining layer of normal synovium represents a paracrine of GDF‐5 to maintain homeostasis of cartilage, however, the redistribution of GDF‐5 expression towards into deeper layers under a chronic pathological conditions in joints suggests a loss in maintenance and repair of intra‐articular joint structure.^84^ In vitro stimulation of TNF caused a reduction in GDF‐5 expression in rheumatoid arthritis and OA fibroblasts compared with unstimulated cells respectively, however, another critical pro‐inflammatory factor, IL‐1β, revealed no change in GDF‐5 expression in OA fibroblasts.^84^ These studies indicate that inflammatory conditions might be the reason why GDF‐5 expression is reduced in OA. However, the aetiology of OA is complex, and whether other pathological factors, such as oxidative stress, cell senescence and nutrition deprivation, can affect GDF‐5 expression requires further investigation.

All these findings indicate that GDF‐5 expression is altered in cartilage with the progression of OA, and the alteration of expression might reflect the function of GDF‐5 and the stage of this disease.

### 5.3 Therapeutic potential of GDF‐5 in OA and cartilage repair

As mentioned above, GDF‐5 is critical for joint homeostasis, especially for cartilage homeostasis. In an in vivo study performed by Parrish et al,^14^ substantial cartilage degeneration and sclerosis of the subchondral bone with formation of bone marrow lesions were observed in a rat model of OA induced by medial meniscus transection (MMT), however, intra‐articular injection of rhGDF‐5 attenuated cartilage lesions on the medial tibial plateau in MMT rats in a dose‐dependent manner. Specifically, single 100 μg rhGDF‐5 injection on day 21 post‐MMT mitigated OA progression with less cartilage matrix degeneration at day 63 post‐MMT, a similar protective effect was observed with two 30 μg injections bi‐weekly.^14^ Interestingly, three 100 μg rhGDF‐5 injections bi‐weekly significantly improved cartilage repair. Therefore, intra‐articular supplementation of rhGDF‐5 could prevent and even reverse OA progression in MMT rats.^14^ These findings provide a rationale for the administration of exogenous Gdf5 to maintain cartilage homeostasis during OA progression.

The possible mechanism of GDF‐5 in cartilage homeostasis could be attributed to its effects on anabolism and catabolism of chondrocytes. A previous study demonstrated that GDF‐5 could maintain the articular chondrocyte phenotype and increase GAG biosynthetic activity in both healthy and OA chondrocytes.^14^ At the molecular level, GDF‐5 stimulated the expression of cartilage anabolic genes *ACAN* and *SOX9* in human chondrocytes.^85^ In addition, IL‐1β is a critical mediator that causes interruption of ECM, characterized by reduced anabolism of the matrix and enhanced catabolism. The inhibition of proteoglycan synthesis of human chondrocytes by IL‐1β could also be markedly counteracted by the treatment with GDF‐5 in alginate bead cultures.^86^ In three‐dimensional in vitro culture, expanded chondrocytes undergoing dedifferentiation lose their chondrocyte phenotype; however, in combination with insulin, GDF‐5 could promote the redifferentiation of expanded chondrocytes and yield cartilaginous constructs.^87^ The β1 integrin family is a major receptor of the ECM,^88^ and decreased expression of α5 integrin, a subunits of β1 integrin family, in articular cartilage is related to chondrocyte dedifferentiation during OA progression.^89^ Interestingly, GDF‐5 can induce the expression of the α5 subunit and the abundant expression of ACAN, collagen type II and Indian hedgehog.^89^ Thus, the chondrocyte phenotype in articular cartilage is preserved because of the presence of GDF‐5, α5 integrin and Indian hedgehog to maintain articular cartilage and prevent hypertrophy.^89^

With respect to catabolism, GDF‐5 supplementation could inhibit the expression of the ECM‐degrading enzymes, including a disintegrin and metalloproteinase with thrombospondin motifs‐4 and matrix metalloproteinase 13 (MMP13) in human chondrocytes. The likely mechanism is that GDF5 stimulation increases the expression of canonical Wnt inhibitors Dickkopf 1 (DKK1) and frizzled‐related protein, thereby inhibiting the canonical Wnt signalling.^85^ Enochson L et al^85^ also indicated that this inhibition was DKK1‐dependent. The DKK1‐mediated inhibition of the canonical Wnt signalling was responsible for the downregulation of MMP13 expression. This finding demonstrates the molecular mechanism of the anti‐catabolic effects of GDF‐5 and could, to some extent, contribute to the understanding of the close link between the GDF‐5 deficiency and OA development.

Osteophytes are a key feature of OA and are associated with pain and functional disability.^90^ Identifying cells that form osteophytes in OA is important to target the cell population for the treatment of OA. It has been shown that platelet‐derived growth factor receptor alpha‐expressing stem/progenitor cells, present in the periosteum and synovium near the articular cartilage, were descendants of the *Gdf5*‐expressing embryonic joint interzone.^91^ These platelet‐derived growth factor receptor alpha + GDF‐5‐lineage cells were activated in OA to form both the cartilage and bone of the osteophyte.^91^ The expanding mineralizing fibrocartilage has been considered an earlier indicator of OA onset.^92^ GDF‐5 progenitors participated in the formation of fibrocartilage, which could develop into osteophytes post‐injury.^92^ GDF‐5 + cells were also found in the enthesis and ligaments, indicating that these GDF‐5 progenitors contribute to tendon enthesis and ligament development.^93^ In addition, these progenitors could produce zonal enthesis following anterior cruciate ligament reconstruction.^94^ The above results suggest that GDF‐5‐lineage cells present fibrocartilage and enthesis where osteophytes commonly occur during OA, and these cell populations could be targeted for the prevention of osteophyte occurrence.

GDF‐5 is also related to cartilage repair and has been well‐studied in this field.^95^, ^96^, ^97^ Katayama et al^98^ reported that the implantation of *Gdf‐5* gene‐transfected autologous BMSCs enhanced repair of full‐thickness articular cartilage defects filled by hyaline cartilage. In another study, GDF‐5 supplementation promoted chondrogenesis and migration of BMSCs in vitro and successfully facilitated the formation of ectopic cartilage with BMSCs in nude mice.^99^ Furthermore, the GDF‐5‐conjugated BMSC‐laden scaffold by 3D‐bioprinting was implanted into the cartilage defect of rats and exerted a better repairing effect on cartilage as well as long‐term chondroprotection compared with the control.^99^ These findings provide a potential biological approach and method that implants cells along with GDF‐5 to repair cartilage defects in OA and other cartilage diseases.

In addition to the supplementation of exogenous GDF‐5, the identification of some molecules or conditions that regulate GDF‐5 signalling and expression also provides new directions for the treatment of OA. For example, recent studies revealed a regulatory mechanism linking Yes‐associated protein (YAP) activity to GDF‐5 expression. YAP was shown to prevent chondrogenic differentiation of MSCs in vitro^100^ and inhibit GDF‐5 expression in chondrogenic MSCs,^101^, ^102^ suggesting a close correlation between YAP and GDF‐5. Furthermore, Kania et al^78^ showed that overexpression of YAP reversed the upregulated expression of GDF‐5. Conversely, YAP knock‐down enhanced GDF‐5 expression in C3H10T1/2 MSCs in micromass cultures, confirming that YAP negatively regulates GDF‐5 expression. They also found that concomitant with downregulated YAP, GDF‐5 was switched on in activated chondroprogenitors in injured synovium, implying that YAP downregulation drives GDF‐5 expression to prime progenitors towards chondrogenesis.^78^

Additionally, SOX11, zinc finger E‐box‐binding homeobox 1 (ZEB1) and paired‐like homeodomain transcription factor 1 (PITX1) are considered candidate molecules that could regulate GDF‐5.^78^ SOX11 could activate GDF‐5 expression because of its direct binding to the 5’‐UTR of the *Gdf‐5*.^79^ Kan et al^79^ further found that SOX11 and GDF‐5 expression were simultaneously decreased in degraded cartilage, both in human samples of OA and in experimental OA mouse model. This finding indicates that SOX11, acting upstream of GDF‐5, could be important for joint homeostasis. For PITX1 and ZEB1, their binding sites are present in the enhancer upstream of the *Gdf‐5* promoter region, which appear to be promising mechanisms for controlling GDF‐5 expression.^103^ However, the potential therapeutic regulation of these three transcription factors needs to be further elaborated.

MicroRNAs (miRNAs), a kind of small endogenous noncoding RNAs, repress gene expression by binding to the 3′‐UTR of target mRNAs. Among the miRNAs implicated in the pathogenesis of OA, miR‐449a and miR‐21, upregulated in human OA cartilage, are highlighted due to their direct modulatory effects on GDF‐5. Overexpression of miR‐21 suppressed the process of chondrogenesis and significantly promoted the levels of catabolic factors by inducing *Gdf‐5* mRNA decay to repress its expression.^104^ Unfortunately, the therapeutic effects of miR‐21 inhibition remain unclear. The upregulated miR‐449a significantly suppressed ECM synthesis and aggravated chondrocyte ECM degradation by inhibiting GDF‐5 expression.^81^ Intriguingly, knock‐down of miR‐449a exhibited the opposite effects with increased mRNA and protein levels of GDF‐5,^81^ which provides an insight that upregulating GDF‐5 via miR‐449a might be a promising therapeutic approach for OA.

**FIGURE 3:**
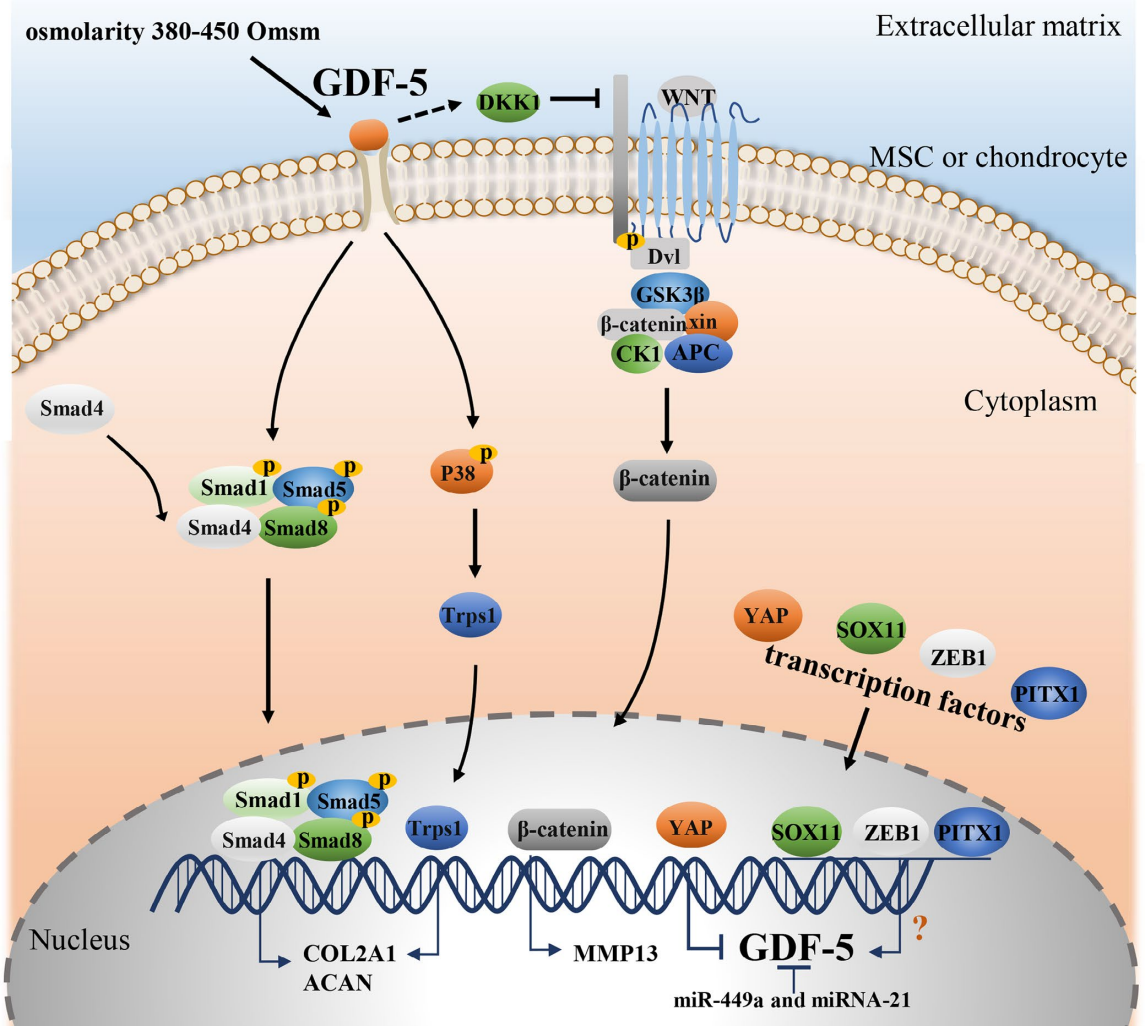
The GDF‐5 signalling pathway and regulation of GDF‐5 expression. GDF‐5, binding to its receptors, activates Smad1/5/8 and subsequently form a complex with Smad 4. The complex then translocates into the nucleus, regulating the expression of target genes, such as *COL2A1* and *ACAN*. In addition, GDF‐5 can induce phosphorylation of p38, which promotes nuclear translocation of Trps1, increasing *COL2A1* gene expression in the chondrogenic cell line (ATDC5). During OA, the activated canonical Wnt signalling pathway induces the expression of the ECM‐degrading enzymes MMP13 in human chondrocytes, whereas GDF‐5 enhances DKK1 to inhibit this signalling. In addition, transcription factors YAP, SOX11, ZEB1 and PITX1 and microRNAs, miR‐449a and miR‐21, regulate GDF‐5 expression. Extracellular osmolarity, ranging from 380 to 450 mOsm, improves cellular responsiveness to GDF‐5

Osmolarity has been demonstrated as a critical parameter of the OA pathobiology, and the proteoglycan loss during OA could reduce the osmolarity of joint tissue to 270 mOsm,^105^, ^106^ whereas the osmolarity of healthy cartilage ranges from 350 to 480 mOsm.^107^ Mang and his colleagues found that increasing the medium osmolarity reduced cytokine release and increased matrix production.^108^ Interestingly, the response of chondrocytes to GDF‐5 at 380 mOsm was more robust than that at 340 mOsm in 3D culture.^108^ These results indicate that osmolarity is involved in OA pathogenesis at least partly by affecting cellular responsiveness to GDF‐5. Nevertheless, modulating osmolarity to improve responsiveness to GDF‐5 in cartilage might be very challenging for OA treatment (Figure 3).

## 6 FUTURE PROSPECTS

Current evidence has indicated that GDF‐5 is essential for the development of cartilage and joints. GDF‐5 possibly functions in a time‐dependent and stage‐dependent manner, but misexpression of GDF‐5 in some critical stages of cartilage development can result in aberrant formation of tissues. Considering the importance and complexity of GDF‐5, more studies are expected to focus on its biological functions in different stages of cartilage and joint development.

In addition, based on extensive studies and meta‐analyses, *GDF‐5* polymorphisms were demonstrated to be highly associated with OA susceptibility, primarily in European and Asian cohorts.^68^, ^109^, ^110^ However, there exists some controversy about this association in some populations, which should be further investigated in broader populations. An exact association of those will partly explain the aetiology of OA at a genetic level. In addition, the molecules or alleles controlling *GDF‐5* expression are potential targets to prevent the onset of OA.

With respect to therapeutic potential, GDF‐5 supplementation shows a significant effect on the chondrogenic process and maintenance of cartilage homeostasis, which provides a promising approach for the treatment of OA and cartilage defects. Moreover, the regulation of GDF‐5 expression also provides some targets to restore the level of GDF‐5 to fulfil its function (Table 1).

**TABLE 1:** GDF‐5 and potential GDF‐5 signalling‐related regulators in cartilage development and OA treatment

GDF‐5/regulator	Target cell/tissue	Target molecule(s)	Main findings	Reference
Recombinant human GDF‐5 (rhGDF‐5)	BMSCs	Smads1‐5‐8	Enhances BMSC chondrogenic differentiation in chondrogenic MSC pellets through increasing incorporation of collagen type II and sulphated GAG into the ECM and promotes the transition of these cultures to hypertrophy and maturation.	39
rhGDF‐5	Foetal human MSCs	—	Improves chondrogenic differentiation.	42
	Human embryonic stem cells			43
	Canine MSCs			44
	Embryonic chick mesenchymal			37
	Rabbits adipose‐derived stromal cells			45
Overexpression of GDF‐5	Adipose stem cells	—	Upregulates COL2A1 and ACAN at the protein and mRNA level and further promotes chondrogenic differentiation.	46
rhGDF‐5	ATDC5 cells	P38/MAPK	Increases the gene expression of COL2A1 and induces chondrogenesis of ATDC cells through activation of p38 signalling pathway.	48
rhGDF‐5	ATDC5 cells	p38 MAPK /Trps1	Promotes the differentiation of ATDC5 cells via p38/Trps1 signalling pathway.	49
rhGDF‐5	Human chondrocytes	—	Increases the GAG biosynthetic activity on both healthy and OA chondrocytes.	14
rhGDF‐5	Human chondrocytes	—	Attenuates IL‐1β‐induced inhibition of proteoglycan (PG) synthesis of human chondrocytes.	86
rhGDF‐5 and insulin	Human chondrocytes	—	Promote the redifferentiation of expanded chondrocytes and yield cartilaginous constructs during 3‐dimensional(3D) in vitro culture.	87
rhGDF‐5	Human chondrocytes	DKK1/Wnt/β‐catenin	Inhibits the expression of the ECM degrading enzymes ADAMTS4 and MMP13 in human chondrocytes by enhancing DKK1 to inhibit the canonical Wnt signalling pathway.	85
rhGDF‐5	Rats cartilage	—	Intra‐articular injection of rhGDF‐5 attenuates cartilage lesions in MMT in a dose‐dependent manner intra‐articular injection of rhGDF‐5 attenuates cartilage lesions in MMT in a dose‐dependent manner.	14
Overexpression of GDF‐5	BMSCs	—	Implantation of GDF‐5 gene‐transfected autologous BMSCs enhanced repair of full‐thickness articular cartilage defects.	98
rhGDF‐5	BMSCs	—	Promotes chondrogenesis and migration of BMSCs in vitro and successfully facilitated formation of ectopic cartilage with BMSCs in nude mice. The implantation of GDF‐5‐conjugated BMSC‐laden scaffold by 3d‐bioprinting into the cartilage defect of rats shows a better repairing effect of cartilage and long‐term chondroprotection.	99
YAP	C3H10T1/2 MSCs	GDF‐5 expression	Overexpression of YAP reverses the upregulated expression of GDF‐5 while knock‐down of YAP enhances GDF‐5 expression in C3H10T1/2 MSCs.	78
SOX11	ATDC5 cells	GDF‐5 expression	SOX11 increases endogenous GDF5 expression by directly binding to the 5’UTR of the GDF‐5 and the expression of SOX11 in company with that of GDF‐5 are decreased in degraded cartilage both in human samples of OA and in mouse experimental OA model.	79
miR‐21	CH8 cells	GDF‐5 expression	miR‐21 suppresses the process of chondrogenesis and significantly promotes the levels of catabolic factors by inducing GDF‐5 mRNA decay to repress its expression.	104
miR‐449a	SW1353 cells	GDF‐5 expression	Upregulated miR‐449a suppresses ECM synthesis and aggravates chondrocyte ECM degradation through inhibiting GDF‐5 expression. Knock‐down of miR‐449a exhibits the opposite effects with increased mRNA and protein levels of GDF‐5.	81
Osmolarity	Human chondrocytes	Responsiveness to GDF‐5	Increasing the medium osmolarity reduces cytokine release and increases matrix production. The response of chondrocyte to GDF‐5 at 380 mOsm is more robust than that at 340 mOsm in 3D culture.	108

Currently, the application of this growth factor is still in infancy. The possible limitations could be speculated as follows. First, GDF‐5, a functional protein, is inclined to serve for local tissues instead to systemic use, which suggests that intra‐articular supplementation or regulation appears to be a more appropriate approach. Second, GDF‐5 may have limited ability to permeate into cartilage because of its size and the effective pore size of ~6 nm of cartilage for molecular diffusion.^111^ Third, human OA chondrocytes respond discordantly to the exogenous GDF5.^112^ All these limitations suggest that single GDF‐5 treatment might not reach its full effects. Accordingly, how to fully exert its anti‐arthritic potential is a critical issue. Cartilage tissue engineering, for example, the combination of GDF‐5 and MSCs for cartilage repair, and the manipulation of the tissue microenvironment allowing chondrocytes to better respond to GDF‐5 provide new insights into the application of this growth factor. Moreover, any method and approach to improve the safety, feasibility and efficacy of GDF‐5 should be considered.

## 7 CONCLUSION

GDF‐5 is essential for cartilage and joint development. *GDF‐5* polymorphisms are significantly associated with OA susceptibility. Importantly, supplementation with GDF‐5 exhibits prominent curative effects in experimental OA, and regulation of GDF‐5 expression and signalling also provides promising therapeutic approaches for this disease. To initially establish this ‘star molecule’ in the clinical treatment of OA, it is necessary to further explore maximizing the availability and efficacy of GDF‐5 in future studies.

## CONFLICT OF INTEREST

The authors confirm that there are no conflicts of interest.

## AUTHOR CONTRIBUTIONS

Fengjing Guo worked on the design and conception of this review. Kai Sun drafted the paper. Jiachao Guo, Xudong Yao and Zhou Guo contributed to revising the paper and all authors approved the final version of the manuscript.

## Data Availability

Data sharing is not applicable to this article as no data sets were generated or analysed during the current study.

## References

[1] Goldring MB . Chondrogenesis, chondrocyte differentiation, and articular cartilage metabolism in health and osteoarthritis. Ther Adv Musculoskel Dis. 2012;4:269‐285. 10.1177/1759720X12448454 PMC3403254 22859926

[2] Michigami T . Regulatory mechanisms for the development of growth plate cartilage. Cell Mol Life Sci. 2013;70:4213‐4221. 23640571 10.1007/s00018-013-1346-9 PMC11113666

[3] Melrose J , Shu C , Whitelock JM , Lord MS . The cartilage extracellular matrix as a transient developmental scaffold for growth plate maturation. Matrix Biol. 2016;54:363‐383. 10.1016/j.matbio.2016.01.008 26807757

[4] Chijimatsu R , Saito T . Mechanisms of synovial joint and articular cartilage development. Cell Mol Life Sci. 2019;76:3939‐3952. 31201464 10.1007/s00018-019-03191-5 PMC11105481

[5] Sophia Fox AJ , Bedi A , Rodeo SA . The basic science of articular cartilage: structure, composition, and function. Sports Health. 2009;1:461‐468. 23015907 10.1177/1941738109350438 PMC3445147

[6] Deng ZH , Li YS , Gao X , Lei GH , Huard J . Bone morphogenetic proteins for articular cartilage regeneration. Osteoarthr Cartilage. 2018;26:1153‐1161. 10.1016/j.joca.2018.03.007 29580979

[7] Glyn‐Jones S , Palmer AJR , Agricola R , et al. Osteoarthritis. Lancet. 2015;386:376‐387. 25748615 10.1016/S0140-6736(14)60802-3

[8] Discher DE , Mooney DJ , Zandstra PW . Growth factors, matrices, and forces combine and control stem cells. Science (New York, N.Y.). 2009;324:1673‐1677. 10.1126/science.1171643 PMC2847855 19556500

[9] Goldring MB , Tsuchimochi K , Ijiri K . The control of chondrogenesis. J Cell Biochem. 2006;97:33‐44. 16215986 10.1002/jcb.20652

[10] Wolfman NM , Hattersley G , Cox K , et al. Ectopic induction of tendon and ligament in rats by growth and differentiation factors 5, 6, and 7, members of the TGF‐beta gene family. J Clin Investig. 1997;100:321‐330. 9218508 10.1172/JCI119537 PMC508194

[11] Pregizer SK , Kiapour AM , Young M , et al. Impact of broad regulatory regions on Gdf5 expression and function in knee development and susceptibility to osteoarthritis. Ann Rheum Dis. 2018;77:450. 29311146 10.1136/annrheumdis-2017-212475 PMC6338229

[12] Loughlin J . Genetic contribution to osteoarthritis development: current state of evidence. Curr Opin Rheumatol. 2015;27:284‐288. 25775188 10.1097/BOR.0000000000000171 PMC4423655

[13] Daans M , Luyten FP , Lories RJU . GDF5 deficiency in mice is associated with instability‐driven joint damage, gait and subchondral bone changes. Ann Rheum Dis. 2011;70:208. 20805298 10.1136/ard.2010.134619

[14] Parrish WR , Byers BA , Su D , et al. Intra‐articular therapy with recombinant human GDF5 arrests disease progression and stimulates cartilage repair in the rat medial meniscus transection (MMT) model of osteoarthritis. Osteoarthr Cartilage. 2017;25:554‐560. 10.1016/j.joca.2016.11.002 27851984

[15] Malinauskas T , Peer TV , Bishop B , Mueller TD , Siebold C . Repulsive guidance molecules lock growth differentiation factor 5 in an inhibitory complex. Proc Natl Acad Sci USA. 2020;117:15620‐15631. 32576689 10.1073/pnas.2000561117 PMC7354924

[16] Storm EE , Huynh TV , Copeland NG , et al. Limb alterations in brachypodism mice due to mutations in a new member of the TGFβ‐superfamily. Nature. 1994;368:639‐643. 8145850 10.1038/368639a0

[17] Hötten G , Neidhardt H , Jacobowsky B , Pohl J . Cloning and expression of recombinant human growth/differentiation factor 5. Biochem Bioph Res Co. 1994;204:646. 10.1006/bbrc.1994.2508 7980526

[18] Chang SC , Hoang B , Thomas JT , et al. Cartilage‐derived morphogenetic proteins. New members of the transforming growth factor‐beta superfamily predominantly expressed in long bones during human embryonic development. J Biol Chem. 1994;269(45):28227‐28234. 7961761

[19] Schreuder H , Liesum A , Pohl J , Kruse M , Koyama M . Crystal structure of recombinant human growth and differentiation factor 5: Evidence for interaction of the type I and type II receptor‐binding sites. Biochem Bioph Res Co. 2005;329:1076‐1086. 10.1016/j.bbrc.2005.02.078 15752764

[20] McDonald NQ , Hendrickson WA . A structural superfamily of growth factors containing a cystine knot motif. Cell. 1993;73:421‐424. 8490958 10.1016/0092-8674(93)90127-c

[21] Fujimura K , Terai Y , Ishiguro N , et al. Heterotypy in the N‐Terminal Region of Growth/Differentiation Factor 5 (GDF5) mature protein during teleost evolution. Mol Biol Evol. 2008;25:797‐800. 18296700 10.1093/molbev/msn041

[22] Heldin CH , Miyazono K , Ten DP . TGF‐beta signalling from cell membrane to nucleus through SMAD proteins. Nature. 1997;390:465‐471. 9393997 10.1038/37284

[23] Massagué J . TGF‐β signal transduction. Annu Rev Biochem. 1998;67:753‐791. 9759503 10.1146/annurev.biochem.67.1.753

[24] Storm EE , Kingsley DM . Joint patterning defects caused by single and double mutations in members of the bone morphogenetic protein (BMP) family. Development. 1996;122:3969. 9012517 10.1242/dev.122.12.3969

[25] Yi SE , Daluiski A , Pederson R , Rosen V , Lyons KM . The type I BMP receptor BMPRIB is required for chondrogenesis in the mouse limb. Development. 2000;127:621.10631182

[26] Nishitoh H , Ichijo H , Kimura M , et al. Identification of type I and type II serine/threonine kinase receptors for growth/differentiation factor‐5. J Biol Chem. 1996;271:21345‐21352. 8702914 10.1074/jbc.271.35.21345

[27] Klammert U , Mueller TD , Hellmann TV , et al. GDF‐5 can act as a context‐dependent BMP‐2 antagonist. BMC Biol. 2015;13:77. 26385096 10.1186/s12915-015-0183-8 PMC4575486

[28] Miyazono K . Signal transduction by bone morphogenetic protein receptors: functional roles of Smad proteins. Bone. 1999;25:91‐93. 10423029 10.1016/s8756-3282(99)00113-1

[29] Park A , Hogan MV , Kesturu GS , et al. Adipose‐derived mesenchymal stem cells treated with growth differentiation factor‐5 express tendon‐specific markers. Tissue Eng Part A. 2010;16:2941‐2951. 20575691 10.1089/ten.tea.2009.0710 PMC2928041

[30] Jimi E , Fei H , Nakatomi C . NF‐κB signaling regulates physiological and pathological chondrogenesis. Int J Mol Sci. 2019;20:6275. 10.3390/ijms20246275 PMC6941088 31842396

[31] Bai Y , Gong X , Dou C , Cao Z , Dong S . Redox control of chondrocyte differentiation and chondrogenesis. Free Radical Bio Med. 2019;132:83‐89. 30394290 10.1016/j.freeradbiomed.2018.10.443

[32] Mackay AM , Beck SC , Murphy JM , et al. Chondrogenic differentiation of cultured human mesenchymal stem cells from marrow. Tissue Eng. 1998;4:415‐428. 9916173 10.1089/ten.1998.4.415

[33] Thomas JT , Lin K , Nandedkar M , et al. A human chondrodysplasia due to a mutation in a TGF‐beta superfamily member. Nat Genet. 1996;12:315. 8589725 10.1038/ng0396-315

[34] Thomas JT , Kilpatrick MW , Lin K , et al. Disruption of human limb morphogenesis by a dominant negative mutation in CDMP1. Nat Genet. 1997;17:58. 9288098 10.1038/ng0997-58

[35] Buxton P , Edwards C , Archer CW , Francis‐West P . Growth/differentiation factor‐5 (GDF‐5) and skeletal development. J Bone Joint Surg Am. 2001;83(Suppl 1):S23‐S30. 11263662

[36] Francis‐West PH , Abdelfattah A , Chen P , et al. Mechanisms of GDF‐5 action during skeletal development. Development. 1999;126:1305. 10021348 10.1242/dev.126.6.1305

[37] Coleman CM , Tuan RS . Functional role of growth/differentiation factor 5 in chondrogenesis of limb mesenchymal cells. Mech Develop. 2003;120:823‐836. 10.1016/s0925-4773(03)00067-4 12915232

[38] Yoo JU , Barthel TS , Nishimura K , et al. The chondrogenic potential of human bone‐marrow‐derived mesenchymal progenitor cells. J Bone Joint Surg Am. 1998;80:1745‐1757. 9875932 10.2106/00004623-199812000-00004

[39] Coleman CM , Vaughan EE , Browe DC , et al. Growth differentiation factor‐5 enhances in vitro mesenchymal stromal cell chondrogenesis and hypertrophy. Stem Cells Dev. 2013;22:1968‐1976. 23388029 10.1089/scd.2012.0282 PMC3685316

[40] Coleman CM , Tuan RS . Growth/differentiation factor 5 enhances chondrocyte maturation. Dev Dynam. 2003;228:208‐216. 10.1002/dvdy.10369 14517992

[41] Murphy MK , Huey DJ , Hu JC , Athanasiou KA . TGF‐β1, GDF‐5, and BMP‐2 stimulation induces chondrogenesis in expanded human articular chondrocytes and marrow‐derived stromal cells. Stem Cells. 2015;33:762‐773. 25377511 10.1002/stem.1890

[42] Bai X , Xiao Z , Pan Y , et al. Cartilage‐derived morphogenetic protein‐1 promotes the differentiation of mesenchymal stem cells into chondrocytes. Biochem Bioph Res Co. 2004;325:453‐460. 10.1016/j.bbrc.2004.10.055 15530414

[43] Oldershaw RA , Baxter MA , Lowe ET , et al. Directed differentiation of human embryonic stem cells toward chondrocytes. Nat Biotechnol. 2010;28:1187‐1194. 20967028 10.1038/nbt.1683

[44] Endo K , Fujita N , Nakagawa T , Nishimura R . Comparison of the effect of growth factors on chondrogenesis of canine mesenchymal stem cells. J Vet Med Sci. 2019;81:1211‐1218. 31167981 10.1292/jvms.18-0551 PMC6715918

[45] Han C , Ren Y , Jia Y , et al. The effective mode of growth and differentiation factor‐5 in promoting the chondrogenic differentiation of adipose‐derived stromal cells. Cell Tissue Bank. 2016;17:105‐115. 26084505 10.1007/s10561-015-9517-6

[46] Feng G , Wan Y , Balian G , Laurencin CT , Li X . Adenovirus‐mediated expression of growth and differentiation factor‐5 promotes chondrogenesis of adipose stem cells. Growth Factors. 2009;26:132‐142. 10.1080/08977190802105917 PMC3034080 18569021

[47] Miyazono K , Kamiya Y , Morikawa M . Bone morphogenetic protein receptors and signal transduction. J Biochem. 2010;147:35‐51. 19762341 10.1093/jb/mvp148

[48] Nakamura K , Shirai T , Morishita S , et al. p38 mitogen‐activated protein kinase functionally contributes to chondrogenesis induced by growth/differentiation factor‐5 in ATDC5 cells. Exp Cell Res. 1999;250:351‐363. 10413589 10.1006/excr.1999.4535

[49] Itoh S , Kanno S , Gai Z , et al. Trps1 plays a pivotal role downstream of Gdf5 signaling in promoting chondrogenesis and apoptosis of ATDC5 cells. Genes Cells. 2008;13:355‐363. 18363966 10.1111/j.1365-2443.2008.01170.x

[50] Holder N . An experimental investigation into the early development of the chick elbow joint. J Embryol Exp Morphol. 1977;39:115. 886251

[51] Shwartz Y , Viukov S , Krief S , Zelzer E . Joint development involves a continuous influx of Gdf5‐positive cells. Cell Rep. 2016;15:2577‐2587. 27292641 10.1016/j.celrep.2016.05.055 PMC4920976

[52] Dy P , Smits P , Silvester A , et al. Synovial joint morphogenesis requires the chondrogenic action of Sox5 and Sox6 in growth plate and articular cartilage. Dev Biol. 2010;341:346‐359. 20206616 10.1016/j.ydbio.2010.02.024 PMC2862098

[53] Polinkovsky A , Robin NH , Thomas JT , et al. Mutations in CDMP1 cause autosomal dominant brachydactyly type C. Nat Genet. 1997;17:18. 9288091 10.1038/ng0997-18

[54] Baur ST , Mai JJ , Dymecki SM . Combinatorial signaling through BMP receptor IB and GDF5: shaping of the distal mouse limb and the genetics of distal limb diversity. Development. 2000;127:605.10631181

[55] Reynard LN , Bui C , Canty‐Laird EG , Young DA , Loughlin J . Expression of the osteoarthritis‐associated gene GDF5 is modulated epigenetically by DNA methylation. Hum Mol Genet. 2011;20:3450‐3460. 21642387 10.1093/hmg/ddr253

[56] Koyama E , Shibukawa Y , Nagayama M , et al. A distinct cohort of progenitor cells participates in synovial joint and articular cartilage formation during mouse limb skeletogenesis. Dev Biol. 2008;316:62‐73. 18295755 10.1016/j.ydbio.2008.01.012 PMC2373417

[57] Decker RS , Koyama E , Pacifici M . Articular cartilage: structural and developmental intricacies and questions. Curr Osteoporos Rep. 2015;13:407‐414. 26408155 10.1007/s11914-015-0290-z PMC4624030

[58] Ray A , Singh PNP , Sohaskey ML , Harland RM , Bandyopadhyay A . Precise spatial restriction of BMP signaling is essential for articular cartilage differentiation. Development. 2015;142:1169‐1179. 25758226 10.1242/dev.110940 PMC4360183

[59] Decker RS , Um H , Dyment NA , et al. Cell origin, volume and arrangement are drivers of articular cartilage formation, morphogenesis and response to injury in mouse limbs. Dev Biol. 2017;426:56‐68. 28438606 10.1016/j.ydbio.2017.04.006 PMC6046638

[60] Miyamoto Y , Mabuchi A , Shi D , et al. A functional polymorphism in the 5' UTR of GDF5 is associated with susceptibility to osteoarthritis. Nat Genet. 2007;39:529‐533. 17384641 10.1038/2005

[61] Köberle B , Koch B , Fischer BM , Hartwig A . Single nucleotide polymorphisms in DNA repair genes and putative cancer risk. Arch Toxicol. 2016;90:2369‐2388. 27334373 10.1007/s00204-016-1771-2

[62] Spector TD , MacGregor AJ . Risk factors for osteoarthritis: genetics11Supported by Procter & Gamble Pharmaceuticals, Mason, OH. Osteoarthr Cartilage. 2004;12:39‐44. 10.1016/j.joca.2003.09.005 14698640

[63] Waarsing JH , Kloppenburg M , Slagboom PE , et al. Osteoarthritis susceptibility genes influence the association between hip morphology and osteoarthritis. Arthritis Rheum. 2011;63:1349‐1354. 21400473 10.1002/art.30288

[64] Valdes AM , Spector TD . The genetic epidemiology of osteoarthritis. Curr Opin Rheumatol. 2010;22(2):139‐143. 20090528 10.1097/BOR.0b013e3283367a6e

[65] Chapman K , Valdes AM . Genetic factors in OA pathogenesis. Bone. 2012;51:258‐264. 22178404 10.1016/j.bone.2011.11.026

[66] Evangelou E , Chapman K , Meulenbelt I , et al. Large‐scale analysis of association betweenGDF5 andFRZB variants and osteoarthritis of the hip, knee, and hand. Arthritis Rheum. 2009;60:1710‐1721. 19479880 10.1002/art.24524 PMC4412885

[67] Richard D , Liu Z , Cao J , et al. Evolutionary selection and constraint on human knee chondrocyte regulation impacts osteoarthritis risk. Cell. 2020;181:362‐381. 32220312 10.1016/j.cell.2020.02.057 PMC7179902

[68] Chapman K , Takahashi A , Meulenbelt I , et al. A meta‐analysis of European and Asian cohorts reveals a global role of a functional SNP in the 5' UTR of GDF5 with osteoarthritis susceptibility. Hum Mol Genet. 2008;17:1497‐1504. 18299287 10.1093/hmg/ddn038

[69] Southam L , Rodriguez‐Lopez J , Wilkins JM , et al. An SNP in the 5′‐UTR of GDF5 is associated with osteoarthritis susceptibility in Europeans and with in vivo differences in allelic expression in articular cartilage. Hum Mol Genet. 2007;16:2226‐2232. 17616513 10.1093/hmg/ddm174

[70] Egli RJ , Southam L , Wilkins JM , et al. Functional analysis of the osteoarthritis susceptibility‐associatedGDF5 regulatory polymorphism. Arthritis Rheum. 2009;60:2055‐2064. 19565498 10.1002/art.24616 PMC6860363

[71] Pan F , Tian J , Winzenberg T , Ding C , Jones G . Association between GDF5 rs143383 polymorphism and knee osteoarthritis: an updated meta‐analysis based on 23,995 subjects. BMC Musculoskel Dis. 2014;15:404. 10.1186/1471-2474-15-404 PMC4265459 25467786

[72] Zhang R , Yao J , Xu P , et al. A comprehensive meta‐analysis of association between genetic variants of GDF5 and osteoarthritis of the knee, hip and hand. Inflamm Res. 2015;64:405‐414. 25894512 10.1007/s00011-015-0818-9

[73] Zhang S , Wang J , Ji H , Jia H , Guan D . Interaction between GDF5 gene polymorphisms and environment factors increased the risk of knee osteoarthritis: a case‐control study. Bioscience Rep. 2019;39:R20182423. 10.1042/BSR20182423 PMC6390126 30777926

[74] Syddall CM , Reynard LN , Young DA , Loughlin J . The identification of trans‐acting factors that regulate the expression of GDF5 via the osteoarthritis susceptibility SNP rs143383. Plos Genet. 2013;9:e1003557. 23825960 10.1371/journal.pgen.1003557 PMC3694828

[75] Reynard LN , Bui C , Syddall CM , Loughlin J . CpG methylation regulates allelic expression of GDF5 by modulating binding of SP1 and SP3 repressor proteins to the osteoarthritis susceptibility SNP rs143383. Hum Genet. 2014;133:1059‐1073. 24861163 10.1007/s00439-014-1447-z PMC4099533

[76] Shin M , Lee S , Kee S , et al. Genetic association analysis of GDF5 and ADAM12 for knee osteoarthritis. Joint Bone Spine. 2012;79:488‐491. 22284607 10.1016/j.jbspin.2011.10.016

[77] Tsezou A , Satra M , Oikonomou P , Bargiotas K , Malizos KN . The growth differentiation factor 5 (GDF5) core promoter polymorphism is not associated with knee osteoarthritis in the greek population. J Orthop Res. 2008;26:136‐140. 17676627 10.1002/jor.20464

[78] Kania K , Colella F , Riemen AHK , et al. Regulation of Gdf5 expression in joint remodelling, repair and osteoarthritis. Sci Rep UK. 2020;10:157. 10.1038/s41598-019-57011-8 PMC6957535 31932746

[79] Kan A , Ikeda T , Fukai A , et al. SOX11 contributes to the regulation of GDF5 in joint maintenance. BMC Dev Biol. 2013;13:4. 23356643 10.1186/1471-213X-13-4 PMC3760452

[80] Bobinac D , Spanjol J , Marinović M , et al. Expression of bone morphogenetic proteins, cartilage‐derived morphogenetic proteins and related receptors in normal and osteoarthritic human articular cartilage. Coll Antropol. 2008;32:83‐87. 19138012

[81] Wu J , Zou M , Ping A , Deng Z , Cai L . MicroRNA‐449a upregulation promotes chondrocyte extracellular matrix degradation in osteoarthritis. Biomed Pharmacother. 2018;105:940‐946. 30021388 10.1016/j.biopha.2018.06.074

[82] Robinson WH , Lepus CM , Wang Q , et al. Low‐grade inflammation as a key mediator of the pathogenesis of osteoarthritis. Nat Rev Rheumatol. 2016;12:580‐592. 27539668 10.1038/nrrheum.2016.136 PMC5500215

[83] Bobacz K , Sunk I , Hayer S , et al. Differentially regulated expression of growth differentiation factor 5 and bone morphogenetic protein 7 in articular cartilage and synovium in murine chronic arthritis: potential importance for cartilage breakdown and synovial hypertrophy. Arthritis Rheum. 2008;58:109‐118. 18163510 10.1002/art.23145

[84] Bramlage CP , Kaps C , Ungethüm U , et al. Modulatory effects of inflammation and therapy on GDF‐5 expression in rheumatoid arthritis synovium. Scand J Rheumatol. 2009;37:401‐409. 10.1080/03009740802120010 18830904

[85] Enochson L , Stenberg J , Brittberg M , Lindahl A . GDF5 reduces MMP13 expression in human chondrocytes via DKK1 mediated canonical Wnt signaling inhibition. Osteoarthr Cartilage. 2014;22:566‐577. 10.1016/j.joca.2014.02.004 24561281

[86] Chubinskaya S , Segalite D , Pikovsky D , Hakimiyan AA , Rueger DC . Effects induced by BMPS in cultures of human articular chondrocytes: comparative studies. Growth Factors. 2009;26:275‐283. 10.1080/08977190802291733 18651287

[87] Appel B , Baumer J , Eyrich D , et al. Synergistic effects of growth and differentiation factor‐5 (GDF‐5) and insulin on expanded chondrocytes in a 3‐D environment. Osteoarthr Cartilage. 2009;17:1503‐1512. 10.1016/j.joca.2009.05.002 19470416

[88] Hynes RO . Integrins: bidirectional, allosteric signaling machines. Cell. 2002;110:673‐687. 12297042 10.1016/s0092-8674(02)00971-6

[89] Garciadiego‐Cázares D , Aguirre‐Sánchez HI , Abarca‐Buis RF , et al. Regulation of α5 and αV integrin expression by GDF‐5 and BMP‐7 in chondrocyte differentiation and osteoarthritis. PLoS One. 2015;10:e127166. 10.1371/journal.pone.0127166 PMC4443976 26010756

[90] Altman R , Asch E , Bloch D , et al. Development of criteria for the classification and reporting of osteoarthritis. Classification of osteoarthritis of the knee. Diagnostic and Therapeutic Criteria Committee of the American Rheumatism Association. Arthritis Rheum. 1986;29:1039‐1049. 3741515 10.1002/art.1780290816

[91] Roelofs AJ , Kania K , Rafipay AJ , et al. Identification of the skeletal progenitor cells forming osteophytes in osteoarthritis. Ann Rheum Dis. 2020;2020‐218350. 10.1136/annrheumdis-2020-218350 PMC8136618 32963046

[92] Dyment NA , Hagiwara Y , Jiang X , et al. Response of knee fibrocartilage to joint destabilization. Osteoarthr Cartilage. 2015;23:996‐1006. 10.1016/j.joca.2015.01.017 PMC4757847 25680653

[93] Dyment NA , Hagiwara Y , Matthews BG , et al. Lineage tracing of resident tendon progenitor cells during growth and natural healing. PLoS One. 2014;9:e96113. 24759953 10.1371/journal.pone.0096113 PMC3997569

[94] Hagiwara Y , Dyrna F , Kuntz AF , Adams DJ , Dyment NA . Cells from a GDF5 origin produce zonal tendon‐to‐bone attachments following anterior cruciate ligament reconstruction. Ann NY Acad Sci. 2019;1460:57‐67. 31596513 10.1111/nyas.14250 PMC6992521

[95] Wu G , Cui Y , Ma L , et al. Repairing cartilage defects with bone marrow mesenchymal stem cells induced by CDMP and TGF‐β1. Cell Tissue Bank. 2014;15:51‐57. 23460257 10.1007/s10561-013-9369-x

[96] Wu G , Cui Y , Wang YT , et al. Repair of cartilage defects in BMSCs via CDMP1 gene transfection. Genet Mol Res. 2014;13:291‐301. 24535856 10.4238/2014.January.17.14

[97] Zhang B , Yang S , Sun Z , et al. Human mesenchymal stem cells induced by growth differentiation factor 5: an improved self‐assembly tissue engineering method for cartilage repair. Tissue Eng Part C Methods. 2011;17:1189‐1199. 21875359 10.1089/ten.tec.2011.0011

[98] Katayama R , Wakitani S , Tsumaki N , et al. Repair of articular cartilage defects in rabbits using CDMP1 gene‐transfected autologous mesenchymal cells derived from bone marrow. Rheumatology (Oxford). 2004;43:980‐985. 15187242 10.1093/rheumatology/keh240

[99] Sun Y , You Y , Jiang W , Zhai Z , Dai K . 3D‐bioprinting a genetically inspired cartilage scaffold with GDF5‐conjugated BMSC‐laden hydrogel and polymer for cartilage repair. Theranostics. 2019;9:6949‐6961. 31660079 10.7150/thno.38061 PMC6815949

[100] Karystinou A , Roelofs AJ , Neve A , et al. Yes‐associated protein (YAP) is a negative regulator of chondrogenesis in mesenchymal stem cells. Arthritis Res Ther. 2015;17:147. 26025096 10.1186/s13075-015-0639-9 PMC4449558

[101] Guo X , Day TF , Jiang X , et al. Wnt/beta‐catenin signaling is sufficient and necessary for synovial joint formation. Gene Dev. 2004;18:2404‐2417. 15371327 10.1101/gad.1230704 PMC522990

[102] Hartmann C , Tabin CJ . Wnt‐14 plays a pivotal role in inducing synovial joint formation in the developing appendicular skeleton. Cell. 2001;104:341‐351. 11239392 10.1016/s0092-8674(01)00222-7

[103] Chen H , Capellini TD , Schoor M , et al. Heads, shoulders, elbows, knees, and toes: modular gdf5 enhancers control different joints in the vertebrate skeleton. PLoS Genet. 2016;12:e1006454. 27902701 10.1371/journal.pgen.1006454 PMC5130176

[104] Zhang Y , Jia J , Yang S , et al. MicroRNA‐21 controls the development of osteoarthritis by targeting GDF‐5 in chondrocytes. Exp Mol Med. 2014;46:e79. 24577233 10.1038/emm.2013.152 PMC3944443

[105] Grushko G , Schneiderman R , Maroudas A . Some biochemical and biophysical parameters for the study of the pathogenesis of osteoarthritis: a comparison between the processes of ageing and degeneration in human hip cartilage. Connect Tissue Res. 1989;19:149. 2805680 10.3109/03008208909043895

[106] Shanfield S , Campbell P , Baumgarten M , Bloebaum R , Sarmiento A . Synovial fluid osmolality in osteoarthritis and rheumatoid arthritis. Clin Orthop Relat Res. 1988;289‐295. 3416536

[107] Urban JP . The chondrocyte: a cell under pressure. Br J Rheumatol. 1994;33:901‐908. 7921748 10.1093/rheumatology/33.10.901

[108] Mang T , Lindemann S , Gigout A . Increasing the medium osmolarity reduces the inflammatory status of human OA chondrocytes and increases their responsiveness to GDF‐5. Int J Mol Sci. 2020;21:531. 10.3390/ijms21020531 PMC7014320 31947660

[109] Capellini TD , Chen H , Cao J , et al. Ancient selection for derived alleles at a GDF5 enhancer influencing human growth and osteoarthritis risk. Nat Genet. 2017;49:1202‐1210. 28671685 10.1038/ng.3911 PMC6556117

[110] Dodd AW , Syddall CM , Loughlin J . A rare variant in the osteoarthritis‐associated locus GDF5 is functional and reveals a site that can be manipulated to modulate GDF5 expression. Eur J Hum Genet. 2013;21:517‐521. 22929025 10.1038/ejhg.2012.197 PMC3641375

[111] DiDomenico CD , Lintz M , Bonassar LJ . Molecular transport in articular cartilage — what have we learned from the past 50 years? Nat Rev Rheumatol. 2018;14:393‐403. 29899547 10.1038/s41584-018-0033-5

[112] Ratnayake M , Plöger F , Santibanez‐Koref M , Loughlin J . Human chondrocytes respond discordantly to the protein encoded by the osteoarthritis susceptibility gene GDF5. PLoS One. 2014;9:e86590. 24466161 10.1371/journal.pone.0086590 PMC3897745

